# Development of a comprehensive, sustained community mental health system in post-earthquake Haiti, 2010–2019

**DOI:** 10.1017/gmh.2019.33

**Published:** 2020-02-11

**Authors:** G. Raviola, A. Rose, J.R. Fils-Aimé, T. Thérosmé, E. Affricot, C. Valentin, S. Daimyo, S. Coleman, W. Dubuisson, J. Wilson, H. Verdeli, G. Belkin, G. Jerome, E. Eustache

**Affiliations:** 1 Harvard Medical School, Boston, MA, USA; 2 Massachusetts General Hospital, Boston, MA, USA; 3 Partners In Health, Boston, MA, USA; 4 University of Maryland, College Park, MD, USA; 5 Zanmi Lasante, Port-au-Prince, Haiti; 6 Mirebalais University Hospital, Mirebalais, Haiti; 7 Teachers College, Columbia University, New York, NY, USA; 8 New York University, New York, NY, USA

**Keywords:** Earthquake, global mental health delivery, Haiti, implementation science, Partners In Health

## Abstract

Between 2010 and 2019 the international health care organization Partners In Health (PIH) and its sister organization Zanmi Lasante (ZL) mounted a long-term response to the 2010 Haiti earthquake, focused on mental health. Over that time, implementing a Theory of Change developed in 2012, the organization successfully developed a comprehensive, sustained community mental health system in Haiti's Central Plateau and Artibonite departments, directly serving a catchment area of 1.5 million people through multiple diagnosis-specific care pathways. The resulting ZL mental health system delivered 28 184 patient visits and served 6305 discrete patients at ZL facilities between January 2016 and September 2019. The experience of developing a system of mental health services in Haiti that currently provides ongoing care to thousands of people serves as a case study in major challenges involved in global mental health delivery. The essential components of the effort to develop and sustain this community mental health system are summarized.

## Background

The Haiti earthquake of 12 January 2010, now at its tenth anniversary, resulted in an estimated 222 000 people killed, 300 500 injured, and more than 1.5 million people homeless (Office for the Coordination of Humanitarian Affairs, 2011). The mental health dimensions of this disaster were massive and reverberated across the country.

In February of 2010, the Minister of Health of Haiti made a direct request to the international health care delivery non-governmental organization (NGO) Partners In Health (PIH) and its local sister organization Zanmi Lasante (ZL) to support the government in developing a functional national mental health system. Recognizing the significant absence of formal mental health services in Haiti prior to the earthquake, PIH sought to address community mental health needs through an approach that both responded to the earthquake emergency immediately, and purposefully established a foundation for greater local capacity to address mental disorders over the long term (Sontag, 2010). An intervention model was developed, starting with a rudimentary theoretical framework called ‘5 by 5’, or 5  *×*  5, to guide the planning of clinical services within the PIH health care system in Haiti's Central Plateau and Artibonite ‘departments’ (regions) (Belkin *et al*., 2011). The experience of developing a system of mental health care in Haiti that currently serves thousands of people provides a case study in the following major challenges related to *the practice of global mental health delivery:* (1) ‘building back better’ mental health services from humanitarian emergencies; (2) developing formal services *de novo* in contexts where mental health services have been minimal; (3) improving treatments and access to care; (4) building and sustaining human resource capacity for mental health service delivery; (5) transforming health system and policy responses; and (6) maintaining funding and momentum for ongoing service delivery in highly resource-constrained contexts, within an area (mental health) that receives minimal development assistance (Collins *et al*., 2011; Epping-Jordan *et al*., 2015).

This case study describes the key conditions relied upon to undertake this ambitious task, which integrated lessons previously learned from emerging implementation literature in global mental health in low-resource settings that often – separately – explores related aspects of overall planning, specific interventions, training, and other components of mental health research and care delivery. These *five key areas of engagement* have included activities that: (I) begin with credible anchor partners to lay a foundation for sustainability; (II) establish an explicit, consensus-driven, strategic framework and Theory of Change (ToC) for system planning; (III) translate these foundations into functional skill packages and care delivery pathways to help people living with conditions that are determined locally to be a priority; (IV) iteratively develop the roles and supervision capability to realize the packages and pathways in the form of sustained care over years; and (V) have an explicit, dedicated approach and capacity for quality improvement (QI), data collection, and change management. A functional mental health health system model is proposed as it has developed to the present, and ten key lessons from this work are summarized.

ZL and PIH were together launched in the mid 1980's in Haiti's Central Plateau with the founding of a small clinic in the village of Cange. PIH and ZL introduced novel programs in Haiti to reduce transmission of, and mortality from, infectious diseases such as human immunodeficiency virus (HIV)/acquired immunodeficiency syndrome (AIDS) and tuberculosis. PIH and ZL collaborated to build a community-based, clinic-supported, hospital-linked health care model capable of delivering high quality services in rural areas without electricity, paved roads or modern sanitation at the time (Farmer *et al*., 2001; Walton *et al*., 2004; Koenig *et al*., 2010). By the time of the 2010 earthquake, ZL was operating 11 government Ministry of Health (*Ministère de la Santé Publique et de la Population*, MSPP) hospitals, employing 5000 staff, many of whom were community health workers (CHWs).

The foundational PIH health care delivery model established in Haiti, since replicated in other countries, was founded on core principles of strengthening primary health care, providing free health care and education, relying on community partnerships, addressing basic economic and social needs, and working in the public sector. ZL's credibility as a local partner working on mental health with the Haitian government was therefore rooted in credibility with the community, significant operational reach geographically, and a real, established care delivery platform founded on principles of respect for context, participation of local stakeholders, and principles of social justice. In 2013, PIH, ZL and the MSPP opened a state-of-the-art hospital, Mirebalais University Hospital (*Hôpital Universitaire Mirebalais*, HUM), on the southernmost border of the Central Plateau nearest to Port-au-Prince, which reinforced the health system's capacity to embed more specialized – as well as community-based – mental health services (Partners In Health, 2014*a*).

Regarding mental health services at ZL prior to the earthquake, from 2005–10 ZL had developed a structured program with a particular focus on HIV-related mental health problems. This program included pre- and post-test HIV counseling, support groups, treatment of depression and anxiety, support for victims of gender-based violence, community education, and referral of people living with severe mental illness to the Mars and Kline University Hospital in Port-au-Prince, the national referral center for mental health services. The ZL Mental Health and Psychosocial Services Program was originally staffed by four dedicated psychologists traveling throughout the Central Plateau and Artibonite departments to ZL-supported facilities and communities. In 2009 PIH inaugurated a formal, nascent mental health program with the purpose of consulting with and linking the organization's ten global sites, so that emerging mental health service delivery platforms being developed at each site would be able to learn from and teach each other, rather than developing in isolation (Smith *et al.*, 2015). Following the earthquake, this pre-existing experience of clinical service delivery led by local experts in Haiti, as well as a pre-existing working relationship between authors EE and GR, served as a foundation for a collaborative ZL and PIH earthquake response that attended to immediate needs while laying a groundwork for longer term health system strengthening (Raviola *et al*., 2012; Raviola *et al*., 2013). A prerequisite for the future success of this ZL-PIH collaboration in mental health was the shared understanding that the highest authority for any final decision-making would always rest with the local team.

### Credible partnerships

A primary function of ZL in Haiti and PIH globally as health care delivery organizations has been to work with communities, government and other partners committed locally to supporting the strengthening of the health system in service to the primary goals and priorities of the Ministry of Health. In addition to communities and government, *partners* can include the World Health Organization and other United Nations agencies, advocacy organizations for mental health, international or local NGOs, academic institutions and individual content experts (clinical, educational and scientific), transnational global health initiatives, transnational corporations, multilateral donor organizations, and private philanthropy. In the setting of disasters the harnessing of effective partnerships can be particularly difficult; however, in the case of Haiti, collaborations not only with the local community and within PIH, but also with other various actors interested in the development of formal mental health services in the country, were essential to the growth of the ZL mental health effort.

The formation of partnerships within Haiti around mental health systems-building presented a significant challenge in the several years following the earthquake, particularly following the disbanding of the UN Cluster formed to coordinate the mental health and psychosocial responses of NGOs working in Haiti in collaboration with MSPP in accordance with the recommendations of the Inter-Agency Standing Committee (Inter-Agency Standing Committee, 2007). In 2011 the Pan American Health Organization (PAHO) carried out an assessment of the Haitian mental health system (World Health Organization/Pan American Health Organization, 2011). The report pointed out that despite the many initiatives of a highly esteemed and dedicated generation of leading Haitian psychiatrists from the 1940's to 60's to establish a mental health sector within MSPP, the country lacked mental health legislation, a national mental health policy, and a mental health strategic plan (World Health Organization/Pan American Health Organization, 2011). Little reliable data on the prevalence of mental health problems were available at the time, and no epidemiological surveillance system was in place. Data on human resources for mental health have been somewhat more available but represent estimates. In 2011 Haiti had between 20 and 27 psychiatrists, most of them in private practice in Port-au-Prince, between 100 and 194 psychologists (including both undergraduate and graduate level), between 50 and 82 social workers, and between 3 and 20 psychiatric nurses working in the country (World Health Organization/Pan American Health Organization, 2011; Nicolas *et al*., 2016). Additionally, an estimated 14 general practitioners had been trained in mental health care, and there was one neurologist in the country (World Health Organization/Pan American Health Organization, 2011). In 2011 mental health services were almost exclusively provided in the public sector at two psychiatric hospitals: the 120-bed Défilé de Beudet Hospital, located just outside Port-au-Prince; and the 60-bed Mars and Kline University Hospital, within Port-au-Prince. The limited government resources available for mental health care delivery were almost entirely centralized within these two facilities. According to the assessment's data, about 18% of patients in these facilities had been hospitalized for 10 years or more. Neither facility had a human rights monitoring system. Besides Mars and Kline, and Beudet Hospitals, some NGOs and religious organizations ran modest mental health programs, particularly in rural areas of the country.

A long history of political turmoil in Haiti has had a negative impact on services across formal and informal institutions, including the healthcare system. The earthquake promoted further erosion of the already fragile structure of the social system. Three presidents have served Haiti from 2015 to the present amid constitutional crises and frequent protests of the population regarding allegations of both electoral fraud and misappropriation of billions of dollars. In 2016 Hurricane Matthew devastated the southern part of the country, with a total of 175 000 people additionally displaced, many facing food insecurity due to widespread damage to crops and livestock, and an ongoing cholera epidemic (Office for the Coordination of Humanitarian Affairs, 2017, 2019). By 2017, 38 000 people were still living in displacement camps established after the 2010 earthquake, not yet resettled or returned to their places of origin (Human Rights Watch, 2017). July 2018 brought new waves of additional street protests, both peaceful and violent, during a new period of *peyi lòk* – country in lockdown – with many people forced by the circumstances to remain at home. Schools were closed, roads were sporadically blocked and the country was brought to a standstill (Charles, 2019; Semple, 2019). As a result, the economy suffered and poverty increased.

In mental health the hope is for partnerships to catalyze change that overcomes such difficulties, as well as various governance challenges that exist in global health delivery, ultimately manifesting as a lack of resources for, and integration of, mental health services in community and primary care, so as to successfully support the development of population-based mental health prevention and care – led by government. A number of engaged, willing partners came forward in Haiti with an interest to support a process that sought to advance a manageable implementation framework and vision for the long-term. Partners showed themselves to be willing to innovate, iterate, and learn over time, along with the implementing team, with an enthusiasm for supporting the building of capacity for mental health care delivery, program management, training and research at ZL, and in the service of the country.

### Strategic framework and theory of change

In general, mental health systems can benefit from the use of explicit strategic frameworks to inform system design. The key assumptions driving the organization of clinical work and implementation of programs from design to practice should be well-matched to the essential task of serving the best interests of people living with mental health problems in context.

With this in mind, in 2010–11 the framework called 5 × 5 was developed by a panel of local and global experts in community mental health who identified *five key skill packages* (or skill sets) for mental health care delivery in low resource settings: (1) case-finding, engagement, follow-up, and psychoeducation; (2) targeted psychological interventions; (3) medication management; (4) supervision and consultation; and (5) quality oversight (Belkin *et al*., 2011). The theoretical framework also included *five key implementation rules* for developing evidence-based pathways and algorithms adapted to context that provided basic guidance on a strategy for change. These included: (1) assess context; (2) identify and map priority care pathways; (3) specify decision support tools; (4) use QI practices; and (5) address sustainability, management, and capacity-building. These were the elements considered fit to the task of addressing the following chronic obstacles to the establishment of mental health care in low-resource settings, including Haiti, and arguably in higher-resource settings as well: too few, or insufficiently trained mental health providers; lack of collaboration or coordination between providers; inadequate supervision; limited proactive identification of cases, engagement, and follow-up; inconsistent diagnosis and matching of evidence-supported treatments to needs; limited integration of local beliefs, practices, and alignment of interventions with community needs; and limited use of methods that capture clinical outcomes to support QI and iterative implementation (Belkin *et al*., 2011).

The initial context assessment, *Implementation Rule 1*, was initiated in March 2011 through a qualitative study in partnership with the Haitian Interuniversity Institute for Research and Development (Interuniversity Institute for Research and Development, 2011), a local social science research initiative, in order to identify local priorities and acceptability of formal services to the community. The assessment engaged 15 focus groups and 11 individual interviews – with community members, CHWs, health workers, people living with mental health problems and their families, Hougan/voudou priests, Manbo/voudou priestesses, and Catholic and Protestant priests – and found that for respondents the stressful conditions related to poverty experienced in many parts of the country, exacerbated by the earthquake, left many people vulnerable to manifestations of sadness and dysthymia, depression, suicide, and psychosis. With regard to mental health services, the opinion consistently expressed across respondents, including from traditional practitioners, was that there was wide acceptance of the need for formal mental health care and services (Interuniversity Institute for Research and Development, 2011). As a result of the initial qualitative study, depression was chosen as an initial condition for the development of an initial care pathway. Over the course of 2011–12, people living with depression, anxiety, traumatic stress, psychotic disorders, epilepsy and other conditions received mental health services of increasing quality and availability. These services were transformational with regard to reducing stigma and changing attitudes related to mental health problems, both in communities as well as within the health system (Partners In Health, 2014*b*). Public education campaigns in communities and on radio also raised awareness of ZL services.

**Fig. 1.**
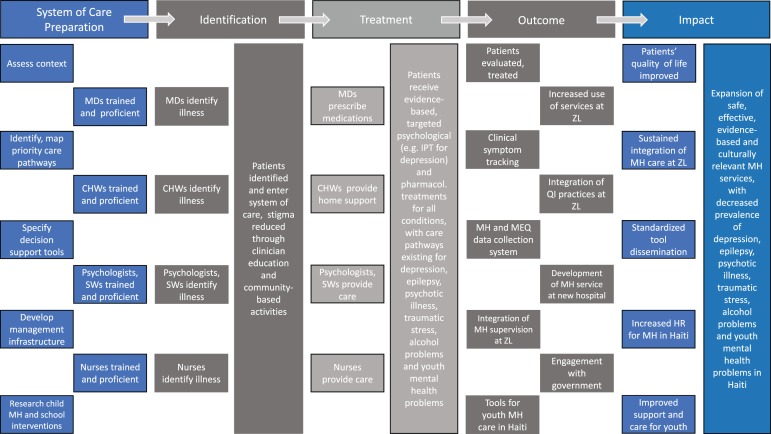
Theory of Change developed in 2012 to guide future mental health systems development.

Having therefore gained experience in the more intensive delivery of mental health care to people living with both severe (i.e. psychotic illness) and common (i.e. depression, anxiety, and stress-related conditions) mental disorders, as well as untreated epilepsy, the collaborative team focused increasingly on establishing the operational, human resource, and content building blocks needed to actualize the 5 × 5 framework, and extend commitments made to develop a functional, model mental health system (Grelotti *et al*., 2015; Legha *et al*., 2015; Fils-Aime *et al*., 2018). In 2012 this led to the development of a *Theory of Change* (ToC), building on the principles embodied in the 5 × 5 framework (Fig. 1). A ToC is a co-created pathway of program design, proposed change and evaluation that starts by making explicit a theory of how a program will achieve its impact, describing the hypothesized steps along a causal pathway, and illustrating the relationships between a variety of outcomes that are each thought of as preconditions for attaining a set of long-term goals (Connell and Kubisch, 1998; Andersen, 2006; Breuer *et al*., 2016). This ToC guided systems planning over the subsequent years, and has been sustained to the present day. Funding support from Grand Challenges Canada beginning in 2012 significantly accelerated stepwise elaboration of the system based upon the ToC, as people living with mental disorders increasingly sought and received care at ZL-supported facilities (Grand Challenges Canada, 2012). The ToC was essential to efforts to coordinate clinical care while the system was in the process of being built. It also aided the engagement of various partners and the evolution of productive partnerships over time, as multiple actors were able to plan for the completion of specific tasks according to a clear vision for growth.

### Skill packages and care pathways

A key building block thread, resting on the growing evidence base for ‘task sharing’ of mental health care components to non-specialist providers with the goal of introducing or increasing access to mental health services, was to first begin by defining the skill packages and competencies that captured the work of evidence-based care pathways, *before* deciding who was to be engaged to do what work (Bolton *et al*., 2003; Patel *et al*., 2011; Singla *et al*., 2017). *Task sharing* in mental health, adapted from prior work in HIV care in low-resource settings, involves the training of non-specialist health workers – individuals with little or no prior formal training or background in mental health care – to deliver mental health care, including community-based support of people living with severe mental disorders, and delivery of brief, low-intensity psychological treatments for people living with common mental disorders (World Health Organization, 2008; Hoeft *et al*., 2018).

At ZL the five *skill packages* were adapted to specific initial clinical pathways (chronologically, (1) *depression*, (2) *epilepsy*, and (3) *psychotic disorders*). Initial training pilots were developed consistent with an initial implementation plan that predated the ToC, predicated on the mobilization of various combinations of people and provider cadres already working in the system (Raviola *et al.*, 2012). While depression was chosen as an initial *care pathway* based on the 2011 qualitative study, epilepsy and psychotic disorders were chosen by the local team based on the prevalence and severity of illness, as well as the clear treatment gap in the community. Increasingly robust training plans for each priority condition, tailored to each cadre of provider (CHWs, social workers and psychologists, nurses, and generalist physicians, respectively) were developed and evolved over the course of 2012–2016. The specific tasks for the care pathways, as they are assigned to each provider currently and which have evolved through real-world clinical practice from 2011 to the present, are summarized in Box 1.

Box 1. Interventions by provider at ZL, 2016–19

**Table:** 

**CHW interventions**
Case identification and referral ZLDSI screening (including for suicidal ideation) Delivery of interventions (IPT-adapted skills, relaxation) Community education (usually around 30 people attending) Community mobilization and sensitization (100+ people) Meetings with community leaders and stigma reduction (usually held by invitation, 10–30 people) Home visits (average 45 minutes per household) Accompany select patients to and from the health facility Waiting with patients at the health facility Follow up with patients in the community Attendance at collaborative care meetings with psychologists (ideally twice a month)
**Psychologist/social worker interventions**
Diagnosis of mental health conditions and neurologic disorders Mental status examination Use screening tools to identify and monitor clinical improvement according to care pathways and protocols – ZLDSI, WHO Disability Assessment Scale (WHODAS), Abnormal Involuntary Movement Scale (AIMS), Clinical Global Impression Scale (CGI), PTSD Checklist (PCL) – Suicidal ideation assessment and safety planning tool Triage of priority mental disorders Recognize and manage medication side effects Provide adherence support Refer patients back to the community Manage QI efforts Delivery of supportive psychotherapy including active and empathic listening, relaxation, and behavioral activation Delivery of manualized, context-adapted psychotherapies – IPT, CBT, CPT Apply specialized knowledge of child and adolescent mental health disorders Respond to mental health crises Refer acute, severe cases to generalist physician Supervise CHW and community activities, including attending meetings with CHWs (ideally twice per month)
**Physician interventions**
As described for psychologists, minus psychotherapy skills Medical evaluation, diagnosis, and follow-up Prescribe psychotropic medications Apply specialized knowledge of neurologic disorders

The initial, system-formative care pathway – depression – was informed by the ‘stepped, collaborative care model’ used in many global settings (Araya *et al*., 2003; Katon, 2012; Coventry *et al*., 2014). The World Health Organization mhGAP Intervention Guide for mental, neurological and substance use disorders in non-specialized health settings, new in 2011, was used as a reference guide for the inclusion in that pathway of interpersonal therapy (IPT) and cognitive-behavioral therapy (CBT) as accepted, evidence-based interventions that could potentially be adapted to the Haitian context (World Health Organization mhGAP, 2011). Both the Haitian and the U.S.-based management teams agreed on the selection of IPT as a potential scalable psychological intervention because of its effectiveness in treating depression and posttraumatic stress-related pathology in high- and low-income settings, as well as its emphasis on the social and interpersonal approach, which was perceived by the local team as critical in Haitian collective society (Verdeli *et al*., 2016).

In order to have *decision support tools* that reflected cultural norms to guide progression along this first depression care pathway, from 2010–11 the team developed a locally valid screening and symptom tracking tool for depression that could be utilized by a range of non-specialist and specialist providers (Rasmussen *et al*., 2015). This tool, the Zanmi Lasante Depression Symptom Inventory (ZLDSI) has been in clinical use across the ZL health system since its development.

Recognizing the challenges facing youth in particular, and the need for more focused research on best practices in care and support to youth in the community, an NIH-funded research study was undertaken to develop a viable school-based mental health intervention in the Central Plateau (Eustache *et al*., 2017). This research also provided the opportunity to validate the use of the ZLDSI in other populations, including for screening of depression in school-going transitional age youth. In 2018, work on a pathway design for trauma and stress-related conditions commenced, utilizing cognitive processing therapy (CPT). Planning is currently underway for a care pathway dedicated to issues related to alcohol use.

### Iterating roles and supervision

Following several years of initial clinical experience in the care of all disorders by a small pilot team, more formal trainings in alignment with the care pathways and protocols began in 2013, starting with depression, followed by epilepsy and psychotic illness. These were rolled out in stages through 2016 based upon when the prior disorder-specific care pathway, curriculum and related encounter tools were completed by the project team, and integrated in the care system, so as to allow time for providers to become comfortable with one care pathway and set of tools before taking on additional knowledge and skills (Partners In Health Toolkit, 2016). All participants were administered pre- and post-tests to measure knowledge transfer. During and after the trainings, input was received from care providers regarding all content, with concerns noted and addressed by the local team, and materials adjusted as needed. This informed *refresher trainings* that were delivered on a cyclical basis from 2015 onwards. Given that staff turnover is an inevitable aspect of global health delivery, the refresher trainings have been necessary both to maintain knowledge and inform practice for providers already working in the system, and also in preparing providers new to the system. In 2019 alone, ongoing, discrete trainings have been completed for CHWs (10), nurses and physicians (7), psychologists (6), traditional healers and community leaders (3), social workers (2), data officers (2), and teachers (2). Formal community engagements have included awareness activities, education, meeting with community leaders, home visits, and referral to additional care.

The skills, tasks, and actions for bringing to life the care pathways has rested largely on bachelor degree-level psychologists, physicians, and CHWs. Iterating, monitoring, and adjusting this distribution of roles, as well as the methods for skill development and coaching of them has occurred in an ongoing fashion, from initial implementation to the present. Perhaps not surprisingly, the 15 bachelor degree-level psychologists working across the ZL clinical sites have borne the greatest burden of the management of the most clinically severe cases. The term ‘task sharing’ has also applied to their practice, given a limited level of initial training and clinical experience prior to their entering the system of care, and the complexity and severity of the cases they see, relative to that training. Learning from and recognizing the specific burdens placed on this care provider cadre in attending to particularly ill patients, a Ph.D.-level supervising psychologist was hired in 2016 to provide additional clinical supervisory support to the psychologists. This Haitian-American clinical supervisor (author CV) was based at the central teaching hospital (HUM), traveled to facilities across the ZL system 3 days per week, and supported front-line care providers for 3 years.

Initially, training for CHWs was developed using a *polyvalent* model in which CHWs would be trained in both mental health care, and in other clinical domains outside of mental health. Feedback from CHWs provided the project team with important, ‘real-world’, practice-based information regarding the lack of clarity of CHW roles within the health system, the perceived burden of pre-existing responsibilities expressed by CHWS, and the fact that providing mental health care was experienced as too burdensome by some. These lessons informed a shift to the training and engagement of *mental health-specific community-based providers* drawn from a specific CHW cadre, the *Agent de Santé Communitaire* (ASC), who have a higher level of pre-existing training than other CHW cadres. Over the course of 2013–14, 25 ASC were formally mobilized as dedicated, mental health-specific CHWs, supervised by the local bachelor degree-level psychologist assigned to oversee their work, and with a defined set of tasks that have expanded with the growth and increasing experience of the ZL team.

Regarding generalist physician supervision in mental health care delivery, in 2011 two generalist physicians were assigned to work in a dedicated way as medication experts and prescribers, as well as trainers of generalist physicians across the ZL system. Ongoing training of generalist physicians has led to their increased engagement in the care of people living with mental disorders. This was initially facilitated in 2011 by the establishment of a fellowship in global mental health delivery shared between PIH and Harvard Medical School (US). The Dr. Mario Pagenel Fellowship in Global Mental Health Delivery honors the life of a cherished ZL physician who perished in the earthquake. The fellowship provides career development for early career U.S. psychiatrists to support local physician training and professional development in psychiatric care at ZL. The fellowship in Haiti has been occupied for 5 out of the past 9 years; however, the absence of a visiting psychiatrist has not precluded the availability of psychiatric supervision and care at ZL given that a number of Haitian generalist physicians have effectively functioned as hospital-based, primary care-oriented, home visiting and prescribing mental health specialists. Efforts to engage nurses on the medical wards in screening for mental health problems and referring patients for care have been less successful.

Overall, the compelling nature of the mental health work at ZL, based on the significant positive response from patients and families in the community as well as the substantial personal and professional development of providers – including opportunities to engage in research, academic presentation and writing, collaborative learning with other PIH global sites, and graduate training in global health delivery – has enabled a high degree of retention of key staff, despite working in particularly difficult circumstances. At times, resistance to taking on new clinical responsibilities, an inevitable aspect of task sharing, has required administrative adjustments for certain cadres of providers. These have included mental health funds supporting a percentage of salaries for physicians and other polyvalent staff (not dedicated solely to mental health-specific tasks), so that the mental health component of the work is more formalized and viewed as a required component of any provider's workflow.

### QI and change management

Developing and adjusting the ensemble of work within a new mental health *care delivery platform* reflected a specific and method-driven commitment to a *change management strategy*, and necessitated the professional development of a *local management and QI team*. This team by necessity took on greater responsibility in administrative management and quality oversight, professional development of front-line providers (e.g. in clinical, teaching, QI and research spheres), leadership development of the growing clinical and management staff, and identification of critical moments at which motivation and retention of staff requires more attention and a greater sense of urgency. Essential work tasks by the collective transnational management team included: care delivery and its expansion; ongoing care pathway development with training and supervision of a range of providers; establishment of monitoring and evaluation infrastructure; and ongoing capacity-building.

Obtaining ‘real-time’ data to support these efforts has evolved and been central to the iterative evolution of the core system. From 2013 to 2015 a paper-based *data collection system* was developed to accompany the new care pathways, tools, and forms, with only aggregate service volume data available electronically. The limited availability of the internet was a significant limiting factor for the more granular collection of data at care delivery sites. In 2016 a facility-based *electronic medical record* (EMR) using Open MRS, not requiring the internet, was piloted at one health center and then implemented gradually over the following months across all 11 ZL facilities. This has enabled patient-level data collection for mental health across the care system.

Initially, psychologists and clinicians working at hospitals and clinics entered data from paper-based clinic files into the OpenMRS EMR at the end of each day. From 2016 to present, at 8 of the 11 facilities the EMR has not regularly connected to the internet. Due to improvements in information technology infrastructure at three of the facilities, the EMR has been consistently connected to the internet at those facilities, and mental health data are integrated with other clinical domains. In 2018 EMR data entry was shifted from psychologists to dedicated *data officers*, which has significantly reduced the burden on the clinicians. However, the long-term goal of the ZL system is to provide more training to providers and to make the OpenMRS platform more user-friendly, in order to facilitate direct entry from providers at the point of care. With the increasing reliability of the facility-based data collection system, the team has been able to record that the ZL mental health system delivered 28 184 patient visits and served 6305 discrete patients at ZL facilities between January 2016 and September 2019.

The elaboration of a data collection system for community-based care, as opposed to facility-based care as described above, has taken more time. In March 2019 ZL shifted from a paper-based CHW data collection system to an electronic system to improve the quality of work of CHWs who screen individuals for mental health disorders, monitor patient symptoms, and complete community stigma reduction activities. The use of CommCare as a community-level data collection tool is now active within all 11 facility catchment areas, and the team is able to collect information at the community level.

QI projects that the facility-based EMR has facilitated have largely centered on supporting the appropriate and clinically indicated use of screening and encounter tools. For example, from EMR data the project team became aware that only the improvement and side-effect domains of the Clinical Global Impression (CGI) Scale were routinely being used by providers, with the third domain of patient severity rarely being assessed. This led to retraining of task-shared mental health staff across health centers on use of the CGI and the importance of all three domains in informing clinical care. Following these retraining efforts, EMR data showed a 21% increase in complete CGI usage from 2017 to 2018 and a further 7% improvement in the use of all CGI domains from 2018 to 2019. The EMR has also been useful in tracking consistency of use of tools such as the ZLDSI, and in identifying variations in practice, as demonstrated by the slight decline in utilization of the ZLDSI in 2019 (Table 1). This has strengthened the ability of the management team to improve usage and practices.

**Table 1.** Tracking of ZLDSI use in the ZL mental health system

Year	% of first visits where a ZLDSI is recorded	% of first visits for patients with depression where a ZLDSI is recorded	% of total visits for patients with depression where a ZLDSI is recorded
2016	61	90	89
2017	62	85	86
2018	60	81	86
2019	62	78	82

Similarly, using EMR data, the project team became aware that one health facility was not routinely scheduling follow-up visits with patients. Following refresher training for providers and data officers at this facility on when, why, and how to schedule follow-up visits for patients, EMR data showed that, as of 2019, the facility was scheduling follow-up visits for 75% of the mental health patients seen. QI efforts such as these, developed collaboratively by the project team, clinical supervisors and clinicians, align with a greater focus on QI in mental health care across PIH global sites from 2018 onward. In 2018 the team also established a clinical dashboard for key mental health indicators that has allowed for a regular feedback loop from data collection at clinical visits, to managers of the service, back to providers, to facilitate the development of projects that aim to improve the quality of care delivered.

The use of proven, systematic methods and tools for change management and QI have generally not received substantial attention within the global mental health field, which has tended to rely on and report on the use of more formal research and evaluation methods. Our experience underscores the primary importance of the former in order to meet ‘real-world’ implementation challenges as they are occurring in sustained practice outside of the scope of research studies, as well as the importance of applying lessons learned in real time by front-line implementing teams. This emphasis includes being very specific in identifying those specific opportunities where more formal research methods have the most potential value, such as the initial context assessment focus work of 2011, the development of the ZLDSI from 2011 to 2012, and in research to inform child pathway design from 2011 to 2014. It highlights the significant challenges facing implementing teams ‘on the ground' in translating strong research evidence to practice, and executing a nuanced set of tasks to advance the primary goal of global mental health – ‘scaling up’ of services through task sharing – in contexts where political will and funding for mental health, trained human resources, and primary care and data collection infrastructure are sparse (Lancet Global Mental Health Group *et al*., 2007; Belkin *et al*., 2011).

**Fig. 2.**
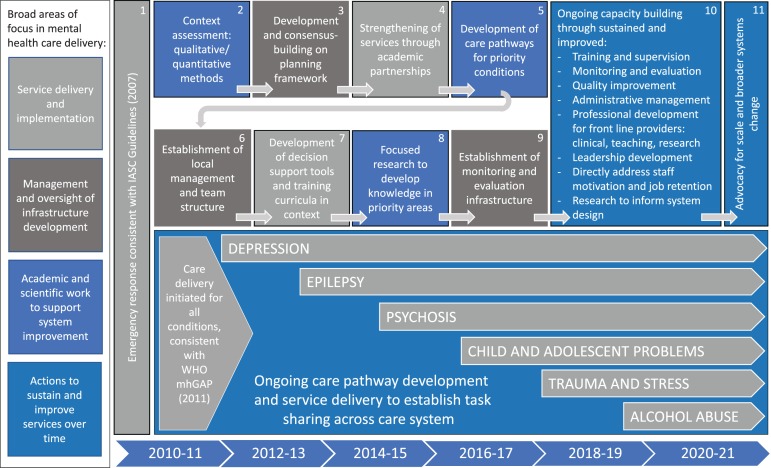
The ZL mental health system model, with broad areas of focus in mental health care delivery, essential system components, and specific care delivery pathways implemented by Partners In Health in Haiti in mental health over the period 2010–19.

Figure 2 brings together the range and sequencing of operational, informational, and evaluative tasks within the broader ToC, for the purpose of achieving comprehensive, sustained service delivery. It summarizes chronologically and sequentially how the *five key areas of engagement* which comprise this case study, informed by the *five key skill packages* and *five key implementation rules* of the original 5 × 5 model, were intentionally brought together over time to clarify the essential system components, sustaining the organized planning of functional clinical care pathways to the present day. The model is colored by four broad areas of thematic focus in mental health care delivery as it actually occurred over a decade: service delivery and implementation; management and oversight of infrastructure development; academic and scientific work to support system improvement; and actions to sustain and improve services over time. The authors propose that this is a credible and unique representation of the ways in which the practice of contemporary, ‘real-world’ global mental health delivery can be conceptualized, not only in Haiti, but also potentially in other low-resource settings globally.

## Summary

Following the 12 January 2010 earthquake in Haiti, and implementing a Theory of Change developed in 2012, PIH and ZL successfully developed a comprehensive, sustained community mental health system in the Central Plateau and Artibonite departments, directly serving a catchment area of 1.5 million people through multiple diagnosis-specific care pathways. The resulting ZL mental health system delivered 28 184 patient visits and served 6305 discrete patients at ZL facilities between January 2016 and September 2019.

### Key lessons

The following lessons and conclusions can be drawn from this work:

(1) *Substantial, measurable delivery of mental health care with a health systems approach in Haiti has been feasible and successful*, despite ongoing political instability. A 2016 consultation from the Pan American Health Organization concluded that the ZL model provides quality services that are culturally adapted, acceptable, and effective in reducing the burden of mental disorders in its catchment area in Haiti, and serves as a viable model for scaling up mental health services at a national level (Pan American Health Organization/Zanmi Lasante/Partners In Health, 2016). The ZL and PIH initiative was also highlighted by Grand Challenges Canada as a success in improving treatments and expanding access for care for mental disorders, through transformational, affordable and cost-effective innovations that have the potential to be sustainable at scale (Grand Challenges Canada, 2019).
(2) *Depression, epilepsy, psychotic disorders, trauma, and substance use problems are treatable in the Haitian context, and benefit from a health systems approach that emphasizes collaboration across provider cadres*. Challenges of collaboration between cadres working within the health system are real, and surmountable. The relative success of task sharing efforts in engaging various cadres of providers has reflected a complex set of variables that deserves further inquiry: the engagement of individual providers with the topic of mental health, based on personal experience and a shared desire to undo the palpable effects of stigma regarding mental disorders that exists in communities and in the health care delivery system itself; the commitment of individual providers' managers to the project of mental health integration; the degree to which providers identified mental health care as a part of their workflow, reinforced by a percentage of their salary being funded specifically for the purpose of mental health service delivery; the nature of the collaborative relationship between providers from a cultural perspective; and the extent to which turnover of staff, as a function of the resource scarcities specific to global health, affects the entire enterprise of task sharing in mental health care delivery. In this work efforts to integrate mental health care have gone beyond acceptance of a simplistic diagnostic, biomedical approach to include the nurturing of functional alliances with local spiritual and religious practitioners, for example with their inclusion in trainings, community education and ongoing outreach.
(3) *The theoretical rudimentary framework – 5 × 5 – effectively outlined the key elements of the care system for future design*. The actual ‘real-world’ model that was developed and has been sustained has contained elements of the original framework. Practical, easily usable system design frameworks such as the one used in this case study serve to keep everyone – care providers, program implementers and managers, collaborators working in allied disciplines across the health system, partnering individuals and organizations, and MSPP colleagues – working toward a shared goal and ‘on the same page'. Such frameworks can potentially serve to advance the practice of global mental health delivery in other global contexts.
(4) *Task sharing that engages non-specialist providers was successful, with expected challenges faced that are representative of broader questions for the field of global mental health in translating research evidence to practice.* Decentralization from health centers to communities through task sharing, and engagement of bachelor degree-level psychologists in provision of supportive psychotherapy and IPT, physicians at health centers in prescription of medications, and mental health-specific CHWs in providing support in the community has been a gradual process that has taken years for the practices to be increasingly accepted and embedded within the existing care system. The success of this work to date in large part resulted from the significant responsibility of managing the care of severely ill people borne by an extremely dedicated cadre of bachelor degree-level psychologists. It must also be acknowledged that the mental health management team, comprised of both ZL and PIH staff, dedicated inordinate time and effort to this work. This sustained commitment seems less feasible in the context of a research study than being embedded within a care delivery system that has committed to maintaining the robust human resource capacity necessary to meet the various needs of people living with mental disorders. A formative PIH organizational value of working according to a *hermeneutic of generosity* – essentially, a commitment to giving others, including colleagues, the benefit of the doubt – rather than a technocratic approach, helped this culturally diverse, interdisciplinary, transnational group to transcend many inevitable challenges to maintain the concept of a cohesive team. Strong team cohesion and positive morale are a *sine qua non* for successful global mental health delivery.
(5) *Training and clinical supervision emerged as one of the two leading challenges faced in the care system*. This has required particular attention in the process of iterative system design. It has also required a very significant human resource commitment and budgeting, including the hiring of full time specialists for this purpose (e.g. an MD-level psychiatrist-fellow, and a Ph.D.-level psychologist, among others). This staffing may not be feasible for many NGOs or even for some ministries of health. Training and supervision, along with human resources for both clinical care and management, have comprised the greatest cost burden for this effort. However, it is the maintenance of refresher training functionality combined with the bolstering of clinical supervisory capacity, as well as striving to constantly improve data collection, that have enhanced scalability within the ZL system of care.
(6) *Monitoring and evaluation of clinical care in mental health, beginning with basic data collection, was identified as the other leading challenge for the care system*. ZL and PIH successfully developed a data collection system and EMR for mental health that effectively facilitate the tracking and improvement of care. It will be essential for care providers to see any electronic system – such as OpenMRS – as a tool to support their work in monitoring patients, and from the beginning to set up feedback mechanisms to inform quality of care, such as clinical dashboards, encounter summary reports, and routine measurement-based QI efforts.
(7) *Monitoring of clinical improvement using locally developed tools has been feasible and favored by the implementing team*. Tracking of the use of tools such as the ZLDSI, CGI, and WHODAS in clinical practice showed a much higher rate of use of the ZLDSI than other tools. This has raised the question of whether its greater acceptability to clinicians, in particular psychologists, was the result of its having been locally developed rather than imported and adapted. The local development of tools, the team's ability to track use of the tools, the tracking of clinical improvement with the tools, and the embedding of the tools within a functional system of mental health care, has been an innovation in Haiti.
(8) *A nimble, adaptive, iterative approach to gradual system change was helpful in steering the change management process.* Effective development and implementation of integrated care pathways and routines, and their successful scale-up within ZL, required ongoing hypothesis-testing, performance data monitoring, and intentional improvement over the course of years. When various unforeseen challenges arose, adaptations were made to the model to continue to advance service delivery, despite tremendous duress borne by the management and care delivery teams. Examples of such adaptations have included changing to monovalent CHWs (dedicated solely to mental health-specific tasks) early on in the system development process, adding in trainings on new topics and refresher trainings on existing topics as needed, revisiting the use of forms or measures that aren't working, and the ongoing improvement of data collection systems and tools.
(9) *Investing in people is essential to the success of the endeavor of global mental health delivery, including equitable salaries for providers, and social support for particularly vulnerable care recipients*. The development of comprehensive, long-term mental health services that link community-based care to primary care, and that integrate traditional perceptions and beliefs with contemporary biopsychosocial approaches (psychosocial, psychological and pharmacologic), requires skilled human beings committed to the difficult but rewarding work of helping people living with mental illness, both severe and common, as well as with related brain disorders such as epilepsy. A long-term commitment outside of a research setting requires a realistic appraisal of the necessary human resource capacity and actual costs for service delivery, clinical supervision, program management, data collection and QI – the collection of activities needed to guarantee a basic quality of care. For example, at ZL in 2016 the full cost of depression care ranged from $19,355 per health center to $25,198 per health center, depending on the amount and intensity of specialist resources and technical support allocated. PIH and ZL, with key partners, bore these specialist and support costs with the knowledge that not making these commitments would place task-shared providers at risk of excessive stress and duress in the delivery of care for severely ill people who have not previously received treatment for debilitating conditions.
(10) *Taking into consideration other efforts in the country to develop mental health programs and services, there exists a substantial foundation for collaborations in mental health care across Haiti*. There have been significant successes elsewhere. Committed partnerships, flexible with regard to contextual challenges and deferential to local belief systems regarding mental illness, will continue to be essential to the successful expansion of this broad collective effort. While there have been many competing priorities over the past decade, since the earthquake some commitment from MSPP to improving mental health system governance has been made (World Health Organization, 2014, 2017). ZL and PIH are committed to supporting the MSPP in continuing to expand public sector mental health care delivery in the country. A proposed psychiatry residency training program at HUM (in addition to the six current and newly ACGME International-accredited medical specialty residencies at the hospital) could support an increase in the number of trained specialists in the country.

### Limitations, challenges, and risks

From the perspective of the service provision reported here, it should be emphasized that the patient visit numbers include only facility-level patients, excluding people cared for in the community. The process of routinely collecting data as it has occurred has also included a large amount of missing data within the data set, and may have resulted in an underestimation of the number of patients seen. Intermittent access to internet and technology challenges, such as computer crashes, have also caused underestimation of patients seen. The actual number of people living with mental health problems cared for by the ZL system is therefore higher than the facility-based numbers presented here.

At a human level, significant challenges currently make service delivery extremely difficult due to the contingencies of the daily reality that providers, coordinators, and managers have been facing over the past decade, a situation that has worsened since 2017. Questions remain about the ongoing viability of a care delivery system that operates within such a highly resource-constrained and politically stressed context. With the increased political and social instability of the past several years, ongoing practical challenges to the work include: reduced availability of clinicians for clinical service duties, related to threats to staff safety, travel restrictions, and other security and safety concerns; fuel shortages, affecting the cost of all items, including transportation fees, by extension limiting patient and clinician transport, patient visits, service use, and the ability of program data officers to facilitate monitoring and evaluation of programs; increasingly underspent grant budgets, due to contextual barriers to completing grant milestones, potentially undermining relationships with donors and other partners; and significant stress and psychological burden to providers delivering care to the population, while attending to their own families and selves.

Despite these challenges, the dedication of various staff across ZL and PIH to maintaining the system and improving the model has led to ongoing program growth and longer-term commitment to build a better system that offers hope that anyone in Haiti living with a mental disorder can have access to free, quality mental health care. We can expect that the perception of the ZL program's relevance as an antidote to despair from mental suffering, both in communities and in the health system, will only increase the demand for services with time.

## Conclusion

As a case study, the shared ZL and PIH effort in Haiti highlights the central importance of detailed planning, partnerships, teamwork, patience and tenacity over time in realizing the dream of sustained, community-based mental health care in low resource settings globally. It informs potential solutions to addressing the Grand Challenges in Global Mental Health regarding improving treatments and expanding access to care: providing effective and affordable community-based care; integrating screening and services into primary care; developing treatments for use by non-specialist providers through task sharing; improving access to medications and reducing costs; improving children's access to evidence-based care; and developing technologies and data collection systems to improve care quality (Collins *et al*., 2011). The adoption of interventions within sustained care delivery systems that reflect the realities of practice ‘on the ground’ (i.e. the bringing together of the science and the practice of implementation) remains a significant challenge for the field of global mental health.

The ZL and PIH mental health teams are hopeful for the ongoing evolution of this work. In particular they credit the ZL workforce and the communities they serve for enthusiastically accepting the challenge of building this system. It is hoped that this work, and its impact, can serve as an effective platform for further advocacy for scale, and broader systems change for mental health in Haiti. *Nap viv e nap kontinye!* – *We are alive, and we will continue!*
